# Fabrication of New Multifunctional Cotton/Lycra Composites Protective Textiles through Deposition of Nano Silica Coating

**DOI:** 10.3390/polym13172888

**Published:** 2021-08-27

**Authors:** Tarek Abou Elmaaty, Hanan G. Elsisi, Ghada M. Elsayad, Hagar H. Elhadad, Khaled Sayed-Ahmed, Maria Rosaria Plutino

**Affiliations:** 1 Department of Material Art, Galala University, Galala 43713, Egypt; 2 Department of Textile Printing, Dyeing & Finishing, Faculty of Applied Art, Damietta University, Damietta 34512, Egypt; hanan.gamal58@yahoo.com; 3 Department of Spinning, Weaving and Knitting, Faculty of Applied Art, Damietta University, Damietta 34512, Egypt; drghada3rm@yahoo.com (G.M.E.); hagarelhadad9@gmail.com (H.H.E.); 4 Department of Agricultural Chemistry, Faculty of Agriculture, Damietta University, Damietta 34512, Egypt; dr_khaled@yahoo.com; 5 Stituto per lo Studio dei Materiali Nanostrutturati, Consiglio Nazionale delle Ricerche, Vill. S. Agata, 98166 Messina, Italy; plutino@pa.ismn.cnr.it

**Keywords:** cotton/lycra composites, silica nanoparticles, antibacterial activity, UV protection, self-cleaning

## Abstract

This study aims to develop multifunctional pile cotton fabrics by implementing different compositions of lycra yarns with different densities of the cotton fabric under study. Highly dispersed silica nanoparticles (SiO_2_ NPs) with small sizes—in the range of 10–40 nm—were successfully prepared and were analyzed using scanning electron microscopy (SEM). The particle size distribution of nano silica was determined via dynamic laser scattering (DLS) and measurements of its zeta potential. Cotton/lycra fabrics were treated using prepared SiO_2_ NPs in presence of ethylenediaminetetraacetic acid (EDTA) as a crosslinking agent. Energy dispersive X-ray (EDX) analysis and scanning electron microscopy (SEM) were used to characterize the nano-treated fabrics and assure homogeneous dispersion of SiO_2_ NPs on the cotton/lycra composites. Additionally, the nanoparticles were screened for their in vitro antibacterial activity against human pathogens such as Gram-positive *Staphylococcus aureus* and *Bacillus cereus* and Gram-negative *Escherichia coli* and *Pseudomonas aeruginosa* strains. The functional properties of the new composite pile cotton fabrics include excellent antibacterial, highly self-cleaning, and excellent UV protection factor (UPF) properties.

## 1. Introduction

Textiles are fundamental to a country’s development and industrialization. As the demand for modern functional textiles grows, new materials and technology are being used. New multifunctional protective and smart textiles have been developed in response to growing technical breakthroughs, new standards, and a customer demand for textiles that are not only attractive but also practical. As a result, high-tech materials and well-considered fabric constructions will enhance wearer comfort while also providing unique features [1]. Textile products made from natural fibers such as cotton are good carriers of a variety of bacteria, which can cause health issues to the wearer [2]. Some of the most serious problems are “skin diseases,” which can be developed by wearing contaminated clothes over a short time with the cross-transmission of bacteria found in air and on ground surfaces. Additionally, presence of these bacteria on textiles can result in undesirable damage to the fabric, such as fading, staining, a reduction in mechanical characteristics, and the material’s deterioration [3].

Cotton is a widely used raw material in the manufacture of many fabrics, especially pile fabrics with many functional properties, such as water absorbency, humidity, durability when wet, and acceptable properties of skin friction, as well as the ability to withstand stress caused by washing and regular use [4]. Pile fabrics have a brush-like surface, which is created by tufts of warp or weft cut threads. A series of threads that protrude at right angles from a foundation or ground structure and form a pile or loop on the surface create the brush-like surface. It differs from other fabrics in regard to the surface texture (loops or cut ends) due to the extra warp and weft threads that appear at a certain height on the surface of the fabric according to the purpose of use, and the pile of these textiles comprises three threads [5]. Pile fabrics are distinguished from other textiles by their ability to illustrate and confirm the functional trends of the fabric’s third dimension, represented by the thickness and height of the pile [6]. Obtaining the pile fabrics constitutes the greatest and most common part of the production of warp pile fabrics. These fabrics consist of two systems of warp threads for weaving warp pile fabrics (pile and ground warp), and one system of weft threads. There are methods of producing warp pile fabrics, including wire pile structures, that are used in upholstery, apparel wear, medical fabrics, ihram outfit clothes, curtains, etc.

However, the use of pure pile cotton fabrics does not achieve the required purpose of comfort and resistance to bacteria, which affects the performance of fabrics, attracts dust, and causes skin problems. Ahmed et al., 2020 [7] endeavored to solve the problems encountered by cotton fabrics using a facile fabrication of multifunctional cotton–modal–recycled aramid blended with protective textiles through the deposition of a three-dimensional tetrakis(hydroxymethyl)phosphonium chloride (THPC)-urea polymer coating. The results exhibited high antibacterial properties and superior water repellency.

On the other hand, synthetic fibers that can be used in the manufacture of pile fabrics such as lycra (Spandex) are seldom used alone. It is stretchy since it is a combination of nylon, cotton, or other fabrics [8]. Further, the use of lycra in woven fabrics provides a superior fit on the body, acting as a second skin, with good form retention and no distortion over the garment’s lifetime. Badr 2017 [2] investigated the influence of fabric structure (Rib 4:1 Plain)/lycra combination on the antibacterial and mechanical properties. The findings revealed that the material type and fabric structure had an impact on the survival of *Escherichia coli* and *staphylococcus aureus* in socks. Lycra’s further characteristics include less moisture absorption and resistance to both sunlight and industrial chemicals. The most common option among the textile is a combination of lycra yarn with different densities to impart a considerable level of stretch and recovery and to improve comfort due to its capacity to stretch, beyond that which can be achieved by cotton alone [9]. The elastomeric properties of a lycra and cotton blend are crucial in determining the elastic product’s end-use [10,11]. Hence, we have focused on improving the functional performance of cotton fabrics by incorporating lycra yarns with different densities.

Performance apparel is exposed to a wide range of external conditions, including sunlight. Different factors promote the growth of melanoma and non-melanoma skin cancers, with UV radiation exposure being one of the most important. As a result, researchers have focused their efforts on altering fabric qualities as a layer to protect the skin from damaging radiation. Modifying the surface of fabrics to protect against UV radiation is crucial [12,13]. The relevance of self-cleaning arises when the fabric is continually exposed to dust and there is not enough time to wash them. One of the advantages of self-clean finishing is the elimination of traditional laundry procedures [14].

Nanotechnology has been successfully applied to various commercial products. It has also gained attention in the textile industry. Currently, it has been used in the processing and finishing of textiles to impart functional benefits [15]. Polymeric nanostructures, metal oxides, carbon nanotubes, clay nanoparticles (NPs), carbon black, graphite nanofibers, and other nanomaterials provide unprecedented textile performance, such as being hydrophilic and hydrophobic, antistatic, wrinkle-resistant, antimicrobial, antiodor, self-cleaning, and antiUV [16,17,18,19,20,21]. Inorganic NPs, especially TiO_2_, ZnO, SiO_2_, Cu_2_O, CuO, Al_2_O_3_, and reduced graphene oxide NPs, are more commonly used than organic NPs due to their thermal and chemical durability at high temperatures, permanent stability under UV rays, and non-toxicity [22,23]. Nano silica has been proven to be a promising material due to its low density and good mechanical stability. Silica (SiO_2_NPs) penetrate easily into a cotton fiber’s interior and attach to the fiber structure tightly. As a result, the hydroxyl group of cellulose and SiOH in SiO_2_NPs form a covalent bond [24,25,26]. This has recently been a significant research topic in both the scientific and industrial sectors. The addition of SiO_2_ NPs to materials’ surfaces increases their mechanical properties and durability, as well as influences their function, activity, and stability [27,28,29].

This study aims to examine the influence of both different fabric structure and implementation lycra yarns on the functional properties of the fabrics, as well as the treatment of the new fabric structure with highly dispersed SiO_2_ NPs. During these processes, ethylenediaminetetraacetic acid (EDTA) was used as a crosslinking agent. It has numerous advantages: it is low cost, locally available, non-toxic, and provides the binding between SiO_2_ NPs and cotton/lycra composites [23,30]. The SiO_2_ treated cotton/lycra composites were also characterized, and their UV protective properties, antibacterial activities, and self-cleaning qualities were measured.

## 2. Materials and Methods

### 2.1. Fabric

The cotton fabric (100%), cotton/lycra (90.8/9.2%), and cotton/lycra (95.5/5.5%) employed have specifications given in Table 1. The weave structure used to prepare the samples was plain weave 1/2. The fabrics were prepared at (Cotton MISR Inc; Almahalla Al-Kubra, Egypt) using an electronic jacquard loom with the specification shown in Table 2. The samples were cleaned for 1 h at a boil with 2 g/L soap and 2 g/L sodium carbonate (Loba Chemie Pvt. Ltd., Boisar, India). The samples were washed in distilled water and air-dried.

### 2.2. Chemicals

Sodium metasilicate (Na_2_SiO_3_._9_H_2_O, 95%, Merk KGaA, Darmstadt, Germany), cetyl trimethyl ammonium bromide (CTAB) (C_19_H_42_BrN, 98%, Loba Chemie Pvt. Ltd., Boisar, India), (HCl) (37%, Merk KGaA, Darmstadt, Germany), ethylene diamine tetra acetic acid (EDTA, Merk KGaA, Darmstadt, Germany), dihydrate, (C_10_H_14_N_2_Na_2_O_8_._2_H_2_O, 99.5%, J.T. Baker, Phillipsburg, NJ, USA), silver nitrate (AgNO_3_, Merk KGaA, Darmstadt, Germany), and sodium chloride (NaCl, Merk KGaA, Darmstadt, Germany) were used in pure forms.

### 2.3. Preparation of Silica Nanoparticles (SiO_2_ NPs)

**Figure 1:**
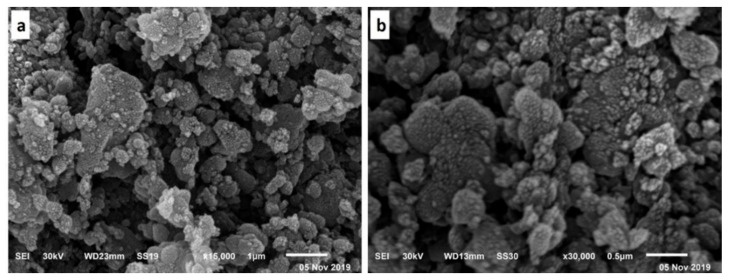
SEM micrographs of the amorphous SiO_2_ NPs at different bar scales (**a**) 1 µm, and (**b**) 0.5 µm.

**Figure 2:**
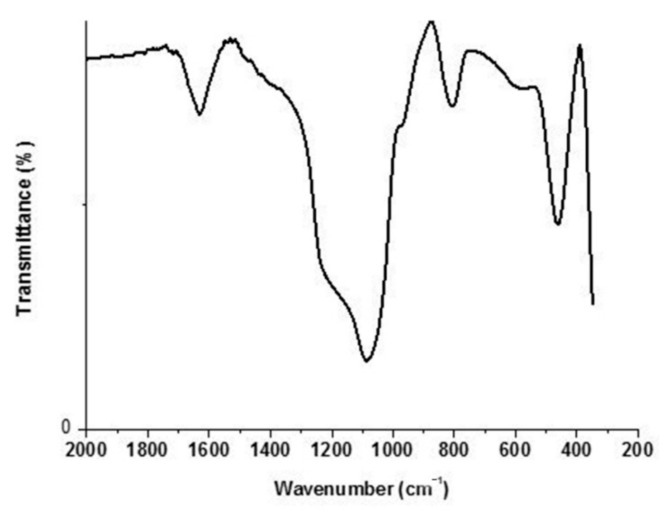
FTIR spectra of the amorphous silica prepared with CTAB.

SiO_2_ NPs were prepared by adding 6 g sodium metasilicate to (394 mL) distilled water in a flask. The temperature was adjusted to 55 °C under constant stirring at a 300 rpm speed. CTAB (4 g) was added with stirring until complete dissolution for 15 min. The diluted HCl (200 mL) was added dropwise to the solution in two steps. In the first step, HCl was added dropwise to the mixture until the pH reached 9–9.5. With no further addition of HCl, the solution was agitated for another 10 min. More HCl was added until the pH reached 3–3.5 in the second step. Then, 10 mL of 10% NaCl aqueous solution was added to the reaction mixture at the acquired pH and agitated for an additional 20 min. The precipitated wet-gel silica was aged at 50 °C for 24 h, and the produced wet-gel silica was centrifuged and rinsed with distilled water until a negative response for chloride ions was observed, as measured with a 0.1 M AgNO_3_ solution. The gel was dried at 40 °C via a microwave heating process. The total drying time was 70 min for each sample, applied seven times in an intermittent interval of 10 min. The dried gel was calcined at 650 °C for 3 h in a muffle furnace to obtain the white powder of nano SiO_2_ [31,32]. The obtained micrographs showed that the SiO_2_ NPs size was in the range of 10–40 nm, whereas their shape was spherical (Figure 1). Moreover, most SiO_2_ NPs had a uniform size, and no aggregation was observed in scanning electron microscopy (SEM) analysis (JEOL JSM-6510LB with field emission gun, Tokyo, Japan). On the other hand, IR spectra were obtained for KBr pellets on a JASCO 410 spectrometer (Jasco, Tokyo, Japan) with only selected absorptions recorded in the range of 2000–200 cm^−1^. Figure 2 shows that the peaks at 1020–1110 and 807 cm^−1^ are attributed to Si-O-Si a symmetric and symmetric stretching style, respectively [33].

#### Dynamic Light Scattering (DLS) and Zeta Potential Analysis

**Figure 3:**
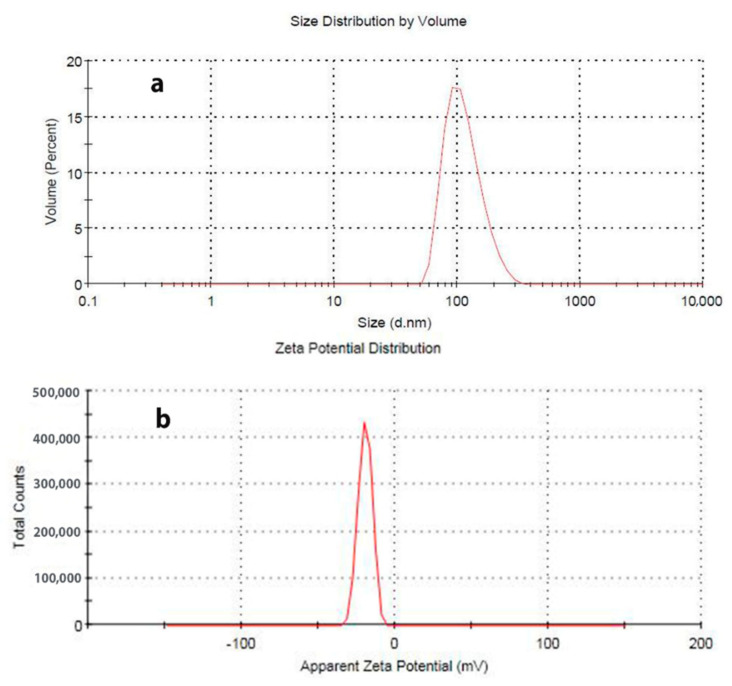
Dynamic light scattering (DLS) (**a**) and zeta potential (**b**) of the obtained SiO2 NPs.

Zeta potential and dynamic light scattering (DLS) were measured using a Malvern Zetasize Nano-zs90. As shown in Figure 3, DLS analysis confirmed the preparation of SiO_2_ particles in nano scale at approximately 90 nm. The obtained size from the DLS analysis is more than that obtained from SEM micrographs, which is caused by the formation of hydrogen bonds between SiO_2_ NPs because of water addition [34]. Furthermore, the zeta potential value of the colloidal SiO_2_ NPs solution was −19.4 mV with a single peak.

### 2.4. Treatment of Fabric with SiO_2_ NPs

The pad-dry-cure procedure was used to incorporate SiO_2_ NPs into the cotton/lycra composites. EDTA (1 g, as a crosslinking agent) was added to 19 mL distilled water to prepare a nano silica solution. Afterwards, the mixture was agitated with a magnetic stirrer. Drops of silica amounting to 0.2 g were added to the liquid and stirred constantly until complete dissolution. The solution was sonicated for 30 min at room temperature. The fabric was soaked in the solution for 60 min followed by drying in an oven at 100 °C until completely dried, then cured at 120 °C for 60 min.

### 2.5. Characterization and Functional Properties of Fabrics Treated with Metal NPs

#### 2.5.1. Scanning Electron Microscopy (SEM) Analysis

The surface morphologies of blank cotton/lycra composites and SiO_2_ NP/cotton/lycra composite fabrics were characterized via scanning electron microscope (JEOL JSM-6510LB with field emission gun, Tokyo, Japan).

The deposition of SiO_2_ NPs into cotton/lycra composites was confirmed using a surface energy dispersive X-ray (EDX) analysis unit (EDAX AMETEK analyzer, Mahwah, NJ, USA) attached to an SEM device. On the other hand, the elemental mapping analysis also reflects the presence of SiO_2_ NPs on the surface of the treated cotton/lycra composites.

#### 2.5.2. Antibacterial Activity

The antibacterial activity of the blank and SiO_2_NP/cotton/lycra composite fabrics against Gram-positive bacteria *Staphylococcus aureus* and *Bacillus cereus* and Gram-negative bacteria *Escherichia coli* and *Pseudomonas aeruginosa* was assessed qualitatively using the AATCC Test Method (147-1988) expressed as the zone of growth (mm) [18,35].

#### 2.5.3. UV Protection Properties

The blank and SiO_2_NP/cotton/lycra composites were examined for UV protection functionality, expressed as UV protection factor (UPF), according to the Australian/New Zealand standard (AS/NZS 4399: 1996), and rated as follows: excellent protection (UPF > 40), very good protection (UPF: 25–39), and good protection (UPF: 15–24) [18,19].

#### 2.5.4. Self-Cleaning

The blank and SiO_2_NP/cotton/lycra composites were tested for self-cleaning by dyeing in a methylene blue dye solution (0.2 g/L) for 5 min and then drying it off. A one-half portion of each stain on the fabric was exposed to sunlight for 24 h to determine their color strength (K/S), whereas the other half portion was covered with black paper to prevent irradiation with sunlight [17]. The K/S value was evaluated using a Spectrophotometer CM-3600A. According to the following equation, decreasing K/S values refer to the level of dye degradation, which is stated as the self-cleaning capacity (SCC):

SCC = [(K/S) b − (K/S) a]/(K/S) b × 100(1)

where (K/S) a: color strength after exposing to daylight, and (K/S) b: color strength before.

## 3. Results and Discussion

A new method for developing high performance functional pile cotton fabric was designed. First, the functional performance of pile cotton fabrics was improved by incorporating a different percentage of lycra yarns with different densities according to the following parameters:

- **Weft material:** Cotton/lycra of six samples: three samples (90.8% cotton and 9.2% lycra), three samples (95.5% cotton and 5.5% lycra), and three cotton samples for the standard comparative.
- **Pile density:** 15, 19, 22 picks/cm.

Second, SiO_2_ NPs were successfully synthesized and then applied on samples under study in a finishing bath containing an aqueous nano-SiO_2_ dispersion and EDTA.

Statistical Analysis

All the data of antibacterial activity and self-cleaning were analyzed using descriptive statistics based on mean and standard deviation.

### 3.1. Characterization of Metal NPs Fabrics

#### 3.1.1. SEM and EDX Analysis

**Figure 4:**
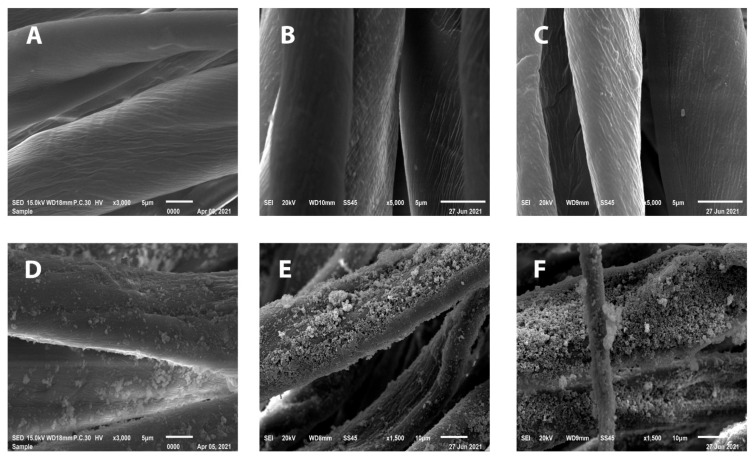
SEM images of (**A**–**C**) untreated samples. SEM images of cotton/lycra (95.5/5.5%) fabric of 15 pick/cm (**D**), cotton/lycra (90.8/9.2%) fabric of 19 pick/cm (**E**), and cotton (100%) fabric of 22 pick/cm (**F**) treated with SiO_2_ NPs.

**Figure 5:**
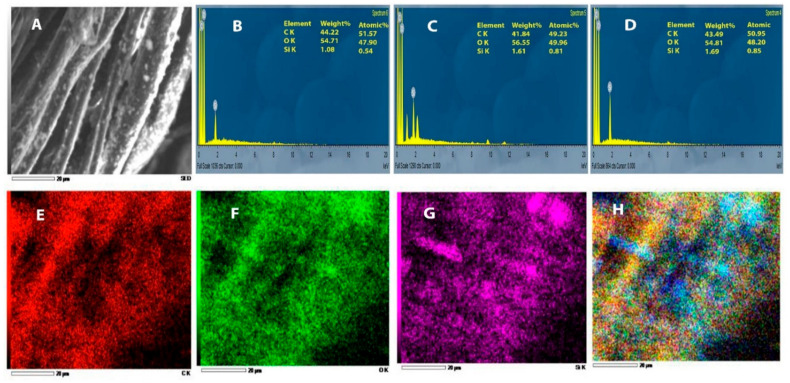
EDX spectrum of (**A**) SEM image (**B**) cotton/lycra (95.5/5.5%) fabric of 15 pick/cm, (**C**) cotton/lycra (90.8/9.2%) fabric of 19 pick/cm, and (**D**) cotton (100%) fabric of 22 pick/cm treated with SiO_2_ NPs, and EDX mapping of cotton/lycra (90.8/9.2%) fabric of 19 pick/cm corresponding to (**E**) carbon, (**F**) oxygen, (**G**) silicon, and (**H**) overlap at 20 µm bar scale.

The SEM images of both untreated cotton/lycra (95.5/5.5%) fabric of 15 pick/cm, cotton/lycra (90.8/9.2%) fabric of 19 pick/cm, and cotton (100%) fabric of 22 pick/cm and cotton/lycra (95.5/5.5%) fabric of 15 pick/cm, cotton/lycra (90.8/9.2%) fabric of 19 pick/cm, and cotton (100%) fabric of 22 pick/cm treated with SiO_2_ NPs are shown in Figure 4A–F, respectively. The results indicated that SiO_2_ NPs are well-distributed on the surface of the fabric, and the presence of SiO_2_ NPs on the fabric surfaces was confirmed from EDX data. The elemental EDX data collected for the fabrics under study were presented in Figure 5. The peaks were allocated at 1.9 keV with 1.69% weight and 0.85% atomic absorption of the analyzed spot in the cotton (100%) fabric of 22 pick/cm, and at 1.9 keV with 1.08% weight and 0.54% atomic absorption of the analyzed spot in the cotton/lycra (95.5/5.5%) fabric of 15 pick/cm, as well as at 1.9 keV with 1.61% weight and 0.81% atomic absorption of the analyzed spot in the cotton/lycra (90.8/9.2%) fabric of 19 pick/cm fiber surface—characteristic of Si. The presence of these peaks confirmed that the SiO_2_ NPs were entirely composed of SiO_2_. The C and O signals originated from the cellulose polymer. Furthermore, the appearance of SiO_2_ NPs on treated fabrics were also investigated by elemental mapping analysis, as illustrated in Figure 5E–G. It is apparent from the mapping images that silicon was distributed well on the surface confirming the uniform coating on cotton/lycra composites using SiO_2_ NPs as a sufficient layer.

#### 3.1.2. Antibacterial Activity of Blank and SiO_2_-NP/Cotton/Lycra Composite Fabrics

**Figure 6:**
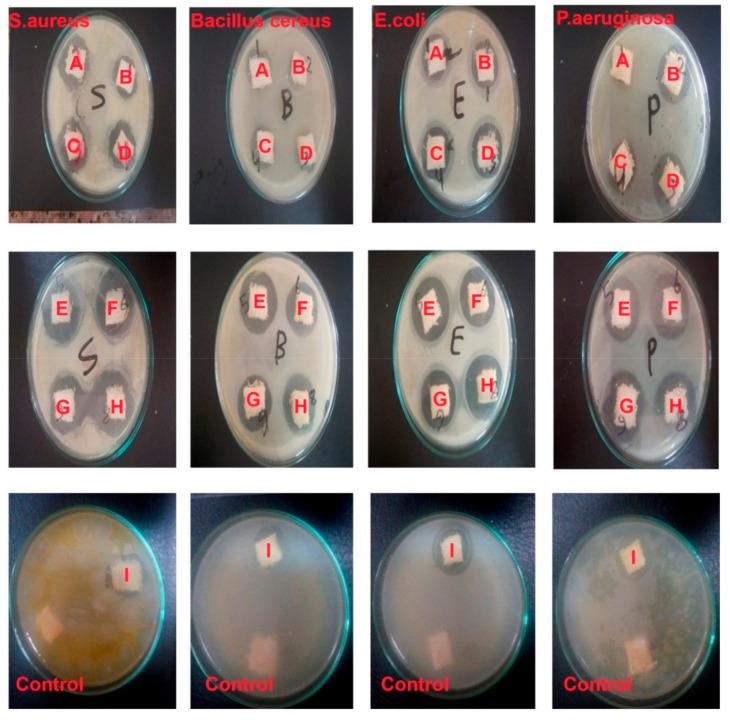
Photograph of Antibacterial Activity of the Blank and SiO_2_ NP/Cotton/Lycra Composites.

The antibacterial properties of treated and untreated samples are summarized in Table 3. Ciprofloxacin is used as a standard to correlate the lead samples from the series (4A–I). It is an antibiotic, causing the production of oxidative radicals and bacterial cell death [36]. The results indicated that the untreated samples were not affected, and there were no inhibition areas. On the other hand, the SiO_2_-NP/cotton/lycra composite fabrics exhibited excellent antibacterial activities against *S. aureus*, *Bacillus cereus*, *E. coli*, and *P. aeruginosa* compared with ciprofloxacin. This may be attributed to the fact that the mode of action of nanoparticles (NPs) is a direct interaction with the bacterial cell wall without the need to penetrate the cell. Most antibiotic resistance mechanisms can enhance the immunity of bacteria causing a lesser response to the antibacterial agent [37]. Samples E and G showed excellent activity against both Gram-positive and Gram-negative bacteria among all treated samples as compared to untreated samples. However, Sample (B) showed good activity against the four model bacteria. Samples (D) and (I) revealed good activity against both Gram-positive and Gram-negative bacteria in comparison to sample (A), which exhibited the lowest activity against Gram-negative (*P. aeruginosa*). Samples (F) and (H) showed good activity against the four model bacteria. However, sample (C) exhibited the lowest activity Gram-positive against (*Bacillus cereus*). In general, Samples (E) and (G) exhibited significantly high antibacterial activity compared to the all other samples in the series (4A–I) and with the standard. Additionally, the density of the pile 19 pick/cm is better than the other densities, as shown in Figure 6. This relies on the fact that there is more trapped air inside this structure (19 pick/cm) than in other densities. This trapped air helps in extending the zone of inhibition around the sample to 19 pick/cm, rendering it more resistant against bacterial attack. SiO_2_ NPs’ extraordinary antibacterial activity is attributed to their large surface area, which allows for better interaction with microbes [29].

#### 3.1.3. UV Protection of Blank and SiO_2_-NP/Cotton/Lycra Composite Fabrics

Table 4 showed the UPF values of the blank and SiO_2_-NP/cotton/lycra composite fabrics. The results illustrated that both the treated and untreated samples have higher values of UPF compared with 100% cotton fabrics. The cover factor was expected to have a favorable impact on UV protection, since traditional cotton fabrics have a lower cover factor than sun protective woven or knitted materials. A main determining component of cover factor is the fabric construction parameter (ends/inch and picks/inch or courses/inch and wales/inch). Because threads are frequently interlaced, woven fabrics offer a larger cover factor than knitted fabrics, as shown in the cotton/lycra) composites under study. The pores between the yarns are small, allowing more radiation to penetrate. The threads in woven cotton/lycra composites are entirely opaque to UV radiation and the pores between the yarns are quite small. UV transmission is proportional to the porosity of the ideal fabric since light can only pass via the pores. The distances between pile fabrics, material thickness, and weight have an impact on the degree of protection. On the other hand, the higher weight and cover factor rate provide 95% of the best protection. The calculation of total UV percent transmittance for a fabric specimen is the ratio of the amount of radiation transmitted to the amount of radiation directed perpendicular to the fabric specimen surface. The cotton/lycra composites generally have no transmission when measuring. The term penetration or erythema weighted transmittance (EWT) is the inverse value of UPF Equation (2) and is frequently used to assign the degree of UVR protection of fabrics. EWT has a range of values between 0 and 1 (or 0 and 100%). The greater the sun protection provided by the fabric, the smaller the percentage of EWT. The results of EWT showed excellent protection (0.0002).

(2) $$\mathrm{EWT}\;=\;\frac{1}{\mathrm{UPF}}$$

The UPF values of SiO_2_-NP-cotton/lycra composite fabrics exhibited excellent UV protection compared to those of blank fabrics, according to the Australian/New Zealand standard. Therefore, these results confirmed the UV refection ability of SiO_2_ NPs, which can effectively decrease aging and reduce human skin damage caused by harmful UV radiation. However, the UPF value of the cotton/lycra (90.8/9.2%) 19 pick/cm is higher than that of the cotton/lycra (94.5/5.5%) 19 pick/cm sample. This may be the result of the finding that, the greater the percentage of lycra in the fabric, the greater the fabric cover factor and, consequently, the greater the UPF values.

#### 3.1.4. Self-Cleaning of Blank and SiO_2_-NP–Cotton/Lycra Composite Fabrics

**Figure 7:**
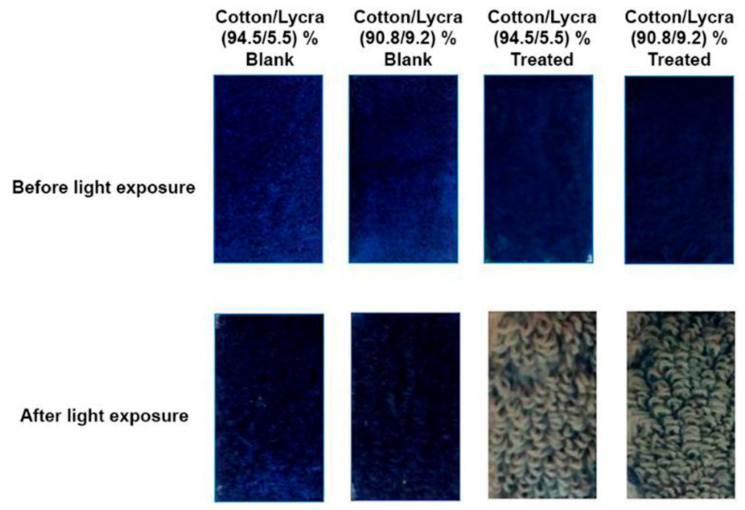
Self-cleaning of the blank and SiO_2_NP/cotton/lycra composite fabrics before and after light exposure.

Table 5 showed that the K/S values of SiO_2_NP/cotton/lycra composite fabrics exposed to sunlight decreased compared to blank fabrics. Compared to the untreated samples, the results of both SiO_2_-NP/cotton/lycra (94.5/5.5%) fabric of 19 pick/cm and cotton/lycra (90.8/9.2%) fabric of 19 pick/cm samples demonstrated greater self-cleaning activity, attributable to the fact that transition metal oxides and their composites, such as SiO_2_ NPs, have strong photocatalytic activity for the photodegradation of organic pollutants, as illustrated in Figure 7. Metal oxide-based nanomaterials with well-controlled structural, crystalline, and surface characteristics behave as semiconductors with broadband gaps and have desirable attributes, including non-toxicity and stability.

## 4. Conclusions

This research demonstrated the effect of both adding lycra yarns with different densities and implementation of SiO_2_ NPs on the functional properties of plain pile cotton fabrics to produce composites. The SiO_2_ NP coated cotton/lycra composites showed significant self-cleaning activity with 24 h of exposure. The self-cleaning results are identical in both the cotton/lycra (94.5/5.5%) and cotton/lycra (90.8/9.2%) 19 pick/cm samples (96.9 and 96.5 values), respectively. Besides the intrinsic antibacterial properties, the inclusion of SiO_2_ NPs will simultaneously lead to the effective antibacterial activity of the fabricated surface. The pile density of 19 pick/cm provides higher bactericidal activity (30–31 mm) compared to other densities. The composite positively influenced the UV protection characteristics compared to blank samples. However, cotton/lycra (90.8/9.2%) 19 pick/cm sample showed higher UPF (4676.8) than did cotton/lycra (94.5/5.5%) 19 pick/cm sample (2690.4). In summary, the cotton/lycra (90.5/9.2%) 19 pick/cm sample showed the best results in terms of antibacterial activity, UV protection, and self-cleaning.

## Figures and Tables

**Table 1:** Specification of the prepared samples.

Fabric Specification	Material	Density/cm	Count (Ne)	Application Method
Warp specification	For ground warp: cotton is used for nine samples For Pile warp: cotton is used for nine samples	24 ends/cm (12 ground ends+12 pile ends)	24/2 Ne for both ground and pile warp ends	Wire pile
Weft Specification	Cotton (100%): for samples (A, B, and C) Cotton/Lycra (90.8/9.2%): for samples (D, E, and F) Cotton/Lycra (95.5/5.5%): for samples (G, H, and I)	(15, 19, 22) picks/cm	16/1 Ne	

**Table 2:** Specifications of the machine used for preparing samples.

Type of Loom	Picanol Nova 600
Manufacturing Country	Italy
Date of Manufacturing	1996
Reed count	12 dent/cm
Denting	2 Ends/Dent
Weft Insertion Device	Rapiers

**Table 3:** Antibacterial Activity of the Blank and SiO_2_NP/Cotton/Lycra Composites. Cotton (100%) 15 pick/cm (**A**), cotton (100%) 19 pick/cm (**B**), Cotton (100%) 22 pick/cm (**C**), cotton/lycra (90.8/9.2%) 15 pick/cm (**D**), cotton/lycra (90.8/9.2%) 19 pick/cm (**E**), cotton/lycra (90.8/9.2%) 22 pick/cm (**F**), cotton/lycra (94.5/5.5%) 19 pick/cm (**G**), cotton/lycra (94.5/5.5%) 22 pick/cm (**H**), cotton/lycra (94.5/5.5%) 15 pick/cm (**I**).

Type of NPs	Sample No.	Zone of Inhibition in mm					
		Gram Positive Bacteria		Gram Negative Bacteria		Mean	SD
		*S. aureus*	*Bacillus cereus*	*E. coli*	*P. aeruginosa*		
Untreated	1	0	0	0	0	0	0
Treated using SiO_2_ NPs	A	22	13	21	5	15.25	7.93
	B	28	12	24	20	21	6.83
	C	28	13	27	28	24	7.35
	D	25	21	21	15	20.5	4.12
	E	30	30	30	26	29	2.00
	F	29	26	30	29	28.5	1.73
	G	29	27	31	31	29.5	1.91
	H	28	25	27	29	27.25	1.71
	I	19	17	23	22	20.25	2.75
Control Ciprofloxacin as antibiotic	J	21	22	26.7	23.3	23.25	2.49

**Table 4:** UPF Values of the Blank and SiO_2_ NP-Cotton/Lycra Composites.

Sample	UPF Values
100% Cotton	30
Untreated Cotton/Lycra (94.5/5.5%) 19 pick/cm	464.9
Treated Cotton/Lycra (94.5/5.5%) 19 pick/cm	2690.4
Untreated Cotton/Lycra (90.8/9.2%) 19 pick/cm	666.7
Treated Cotton/Lycra (90.8/9.2%) 19 pick/cm	4676.8

Note. UPF = UV protection factor.

**Table 5:** Self-cleaning of the blank and SiO_2_-NP/cotton/lycra composite fabrics.

Sample	K/S Value Unexposed	SD	K/S Value Exposed	SD	SCC
Blank	25.8	0.32	11.9	0.25	53.8
Cotton/Lycra (94.5/5.5%) 19 pick/cm	20.7	0.31	0.63	0.02	96.9
Blank	25.3	0.32	16.7	0.33	33.9
Cotton/Lycra (90.8/9.2%) 19 pick/cm	21.1	0.23	0.73	0.03	96.5

Note. K/S = (Color strength).

## Data Availability

The data presented in this study are available on request from the corresponding author.
